# Endoscopic ligation technique for refractory gastrotracheal fistula

**DOI:** 10.1002/deo2.320

**Published:** 2023-11-22

**Authors:** Hironari Shiwaku, Akio Shiwaku, Hiroki Okada, Hiroshi Kusaba, Kenji Maki, Hideki Shimaoka, Kosuke Yamauchi, Yasuhiro Hashimoto, Teppei Yamada, Fumihiro Yoshimura, Suguru Hasegawa

**Affiliations:** ^1^ Department of Gastroenterological Surgery Fukuoka University Faculty of Medicine Fukuoka Japan

**Keywords:** endoscopic closure, endoscopic ligation technique, endoscopic suturing, extracorporeal ligation, gastrotracheal fistula

## Abstract

Endoscopic therapy has recently undergone remarkable progress, including the use of suturing procedures within the gastrointestinal tract using flexible endoscopes. However, existing suturing techniques primarily involve closure using instruments or continuous sutures using an endoscopic needle holder, leaving a gap in nodal suturing methods with extracorporeal ligation. This paper introduces a novel approach, the endoscopic ligation technique, wherein a flexible endoscope is utilized for nodal suturing through extracorporeal ligation.

## INTRODUCTION

With the advancement of endoscopic therapy, an increasing number of studies are reporting on suturing within the gastrointestinal tract lumen using a flexible endoscope. However, to date, suturing techniques have mainly involved closure using instruments (clips and closure devices) or continuous suturing using an endoscopic needle holder.^1^, ^2^, ^3^ No reports have described the placement of nodal sutures with extracorporeal ligation using a flexible endoscope. In this report, we present a novel endoscopic ligation technique (ELT) with extracorporeal ligation using a flexible endoscope.

## CASE REPORT

### ELT method

**FIGURE 1:**
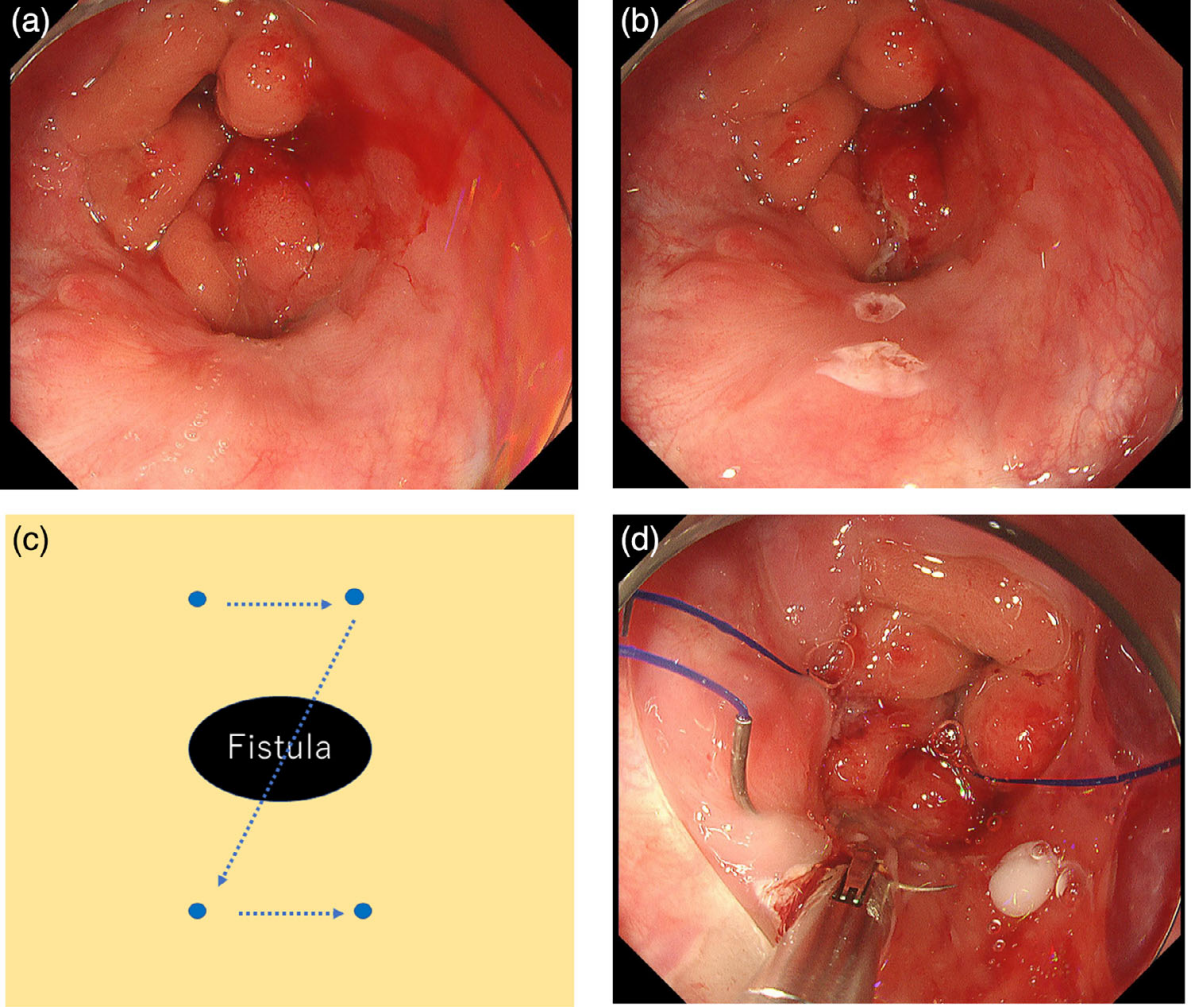
Overview of the endoscopic ligation technique (procedures inside the body). (a) The patient is positioned so that the fistula is at 6 o'clock. (b) Markings are placed on the anal and oral sides of the fistula to ensure easy identification. (c) While longitudinal suturing is desirable to prevent the narrowing of the gastrointestinal tract, it is not feasible with the current instruments for suturing and endoscopy in narrow luminal structures like the esophagus or stomach. Therefore, a Z‐stitch is placed so that the direction of ligation follows the longitudinal axis of the intestines. (d) Implementation of the Z‐stitch. The needle passes deep into at least the deep submucosal layer to achieve secure ligation.

The procedure was carried out under general anesthesia with CO_2_ insufflation. For suturing with the endoscopic needle holder, the position was adjusted so that the fistula was located at 6 o'clock (Figure 1a).

**FIGURE 2:**
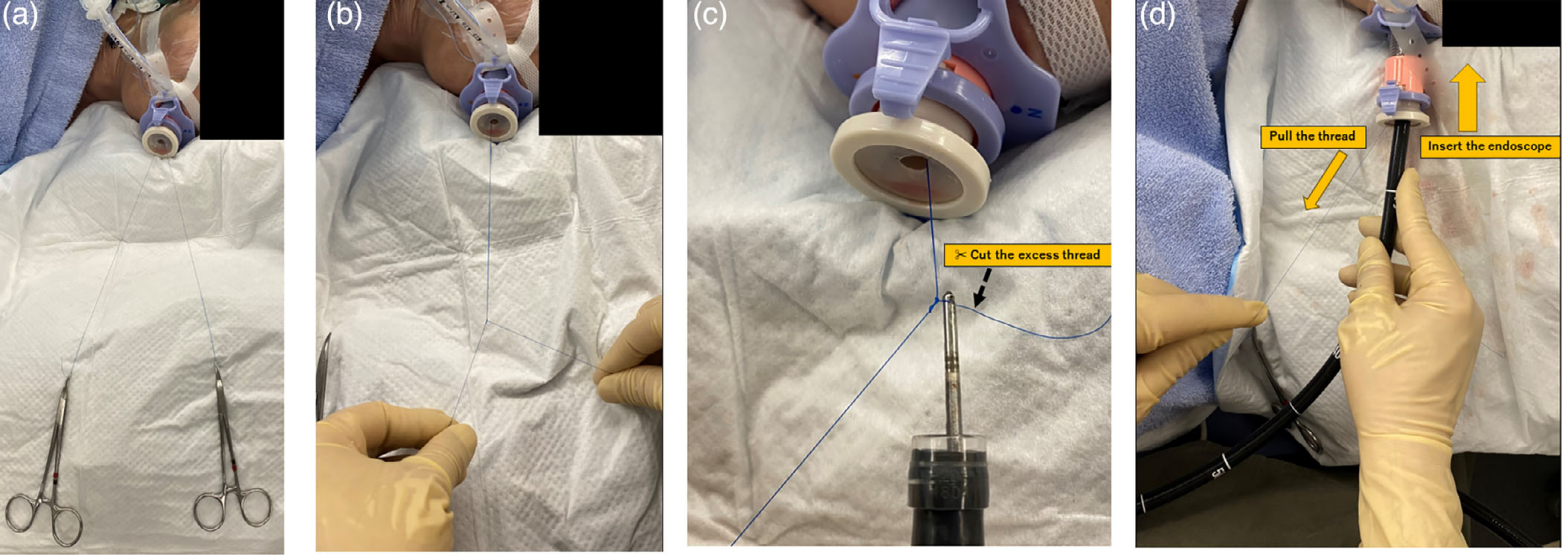
Overview of the endoscopic ligation technique (procedures outside the body). (a) Both ends of the thread are extended outside the body (b) Create extracorporeal knotting. (c) The thread is firmly grasped with the needle holder brought out from the forceps hole, and any excess thread is cut. (d)The surgeon's left hand pulls the thread, while the right hand inserts the endoscope and guides the knot through the overtube to the fistula location.

An endoscope (GIF‐H290T; Olympus) with a transparent hood attachment (D‐201‐11804; Olympus) was used. The distance to the fistula was measured and an overtube of appropriate length to avoid contact with the fistula was chosen and inserted. As the fistula was not easily distinguishable when approached tangentially, both the anal and oral sides of the fistula were marked using argon plasma coagulation (Figure 1b). A threaded needle (4‐0 Prolene 90 cm, 13 or 17 mm, 1/2 round, Ethicon, D4295 or 8357H) was affixed to an endoscopic needle holder (FG‐260Q; Olympus), and a Z‐shaped suture (full‐layer sutures or sutures that reach at least to the deep submocosal layer) was placed from the anal to the oral side (Figure 1c,d). The two ends of the thread were brought outside the body and after confirming with the anesthesiologist that the endotracheal tube was not immobilized or that the balloon was not damaged by the full‐layer sutures, extracorporeal knotting was performed. (Figure 2a,b). The thread was securely held using an endoscopic needle holder through the forceps hole of the endoscope, and any excess thread was trimmed (Figure 2c). The surgeon's left‐hand pulls the thread, while the right‐hand inserts the endoscope and guides the knot through the overtube to the fistula location (Figure 2d). An assistant manipulated the angle of the endoscope to guide the suture node to the intended site. The ligations were firm, akin to those used in typical extracorporeal ligation during laparoscopic surgery, ensuring secure fistula closure (Supporting Information).

### Clinical cases

The ELT was applied in two patients with gastrotracheal fistula. Written informed consent was obtained from both patients. The treatment was in accordance with the principles of the Declaration of Helsinki.

#### Case 1

A 54‐year‐old woman had undergone subtotal esophagectomy for esophageal cancer in a previous hospital. Two weeks after esophagectomy with gastric tube reconstruction, she developed a gastrotracheal fistula due to anastomosis leakage. Several unsuccessful attempts were made to endoscopically close the fistula using clips and sheets. Consequently, she developed a chronic cough and was unable to eat, necessitating central intravenous nutrition for 3 months. Despite her previous doctor recommending surgical closure of the fistula, she requested endoscopic closure and was referred to our department.

**FIGURE 3:**
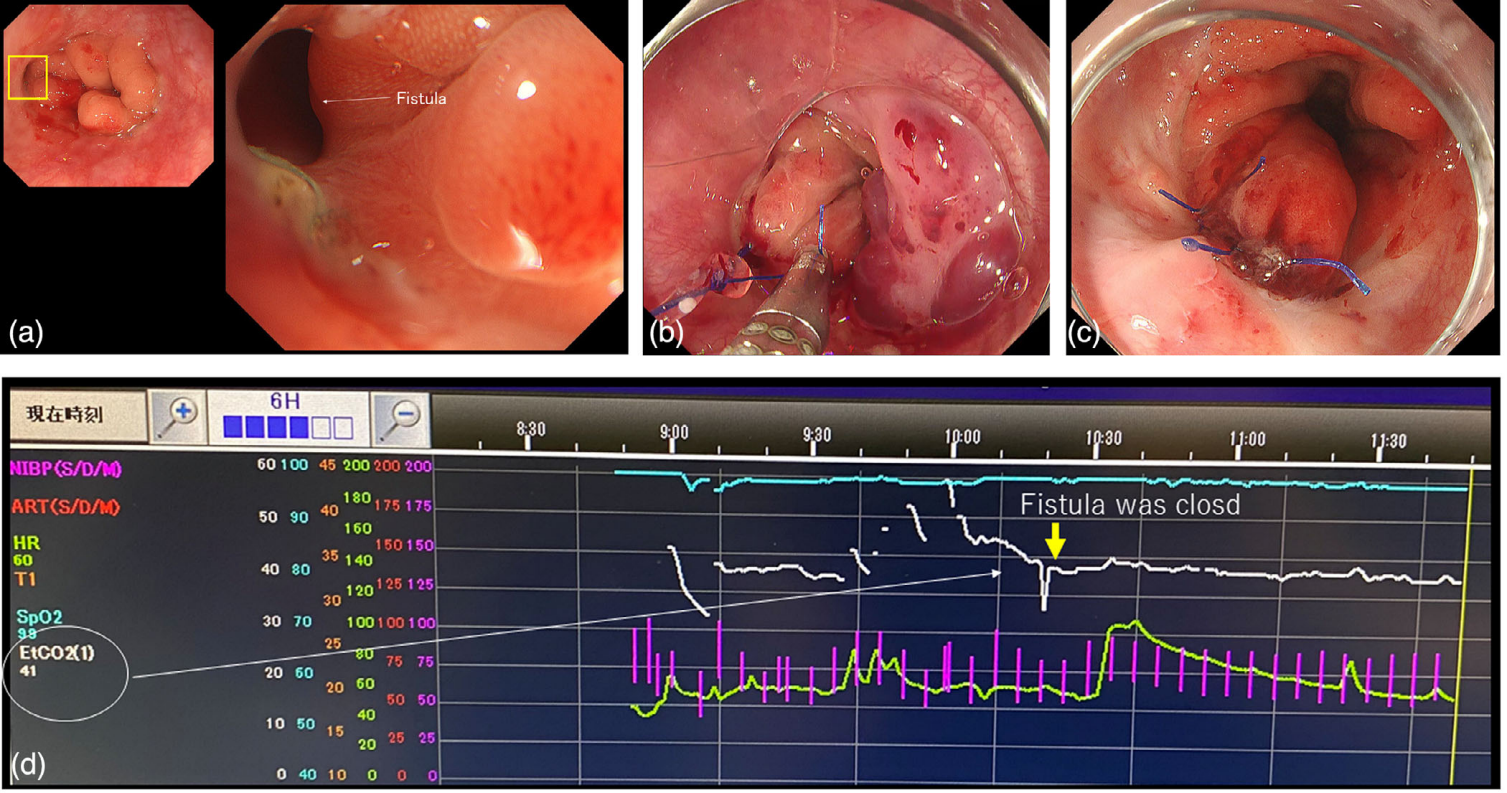
Images from Case 1 in which fistula closure was not achieved with the endoscopic ligation technique. (a) A 3‐mm fistula opening at the anastomosis site. Tracheal wall observed from the gastrointestinal lumen. (b) The knotted portion of the thread is advanced to the ligation site under endoscopic visualization. Secure ligation is performed similarly to conventional extracorporeal suturing during laparoscopic surgery until the fistula is firmly closed. (c) Two stitches are placed using the endoscopic ligation technique. (d) The EtCO2 is increased before fistula closure but returned to normal after fistula closure.

A 3‐mm gastrotracheal fistula was identified in the anterior wall (Figure 3a). The fistula was closed with two sutures using the ELT (Figure 3b,c). The EtCo2 was elevated before the closure of the fistula but normalized after the fistula closure (Figure 3d). Although the closure was maintained, her cough recurred after 1 month. Endoscopic examination revealed wound separation. A bronchotomy was performed and direct full‐layer suturing was attempted from the tracheal side, but fistula closure could not be achieved. A covered stent has been placed in the fistula, and the patient is currently awaiting surgery.

#### Case 2

A 44‐year‐old man underwent subtotal esophagectomy for esophageal cancer 1 year ago. On postoperative day 6, he had a minor leakage, which resolved with conservative treatment. Six months postoperatively, he developed a cough on food intake. A 2‐mm esophageal‐pulmonary fistula was detected. Attempts to achieve closure using clips were unsuccessful. The patient developed a chronic cough and was unable to eat. He requested endoscopic fistula closure, and the ELT was performed.

**FIGURE 4:**
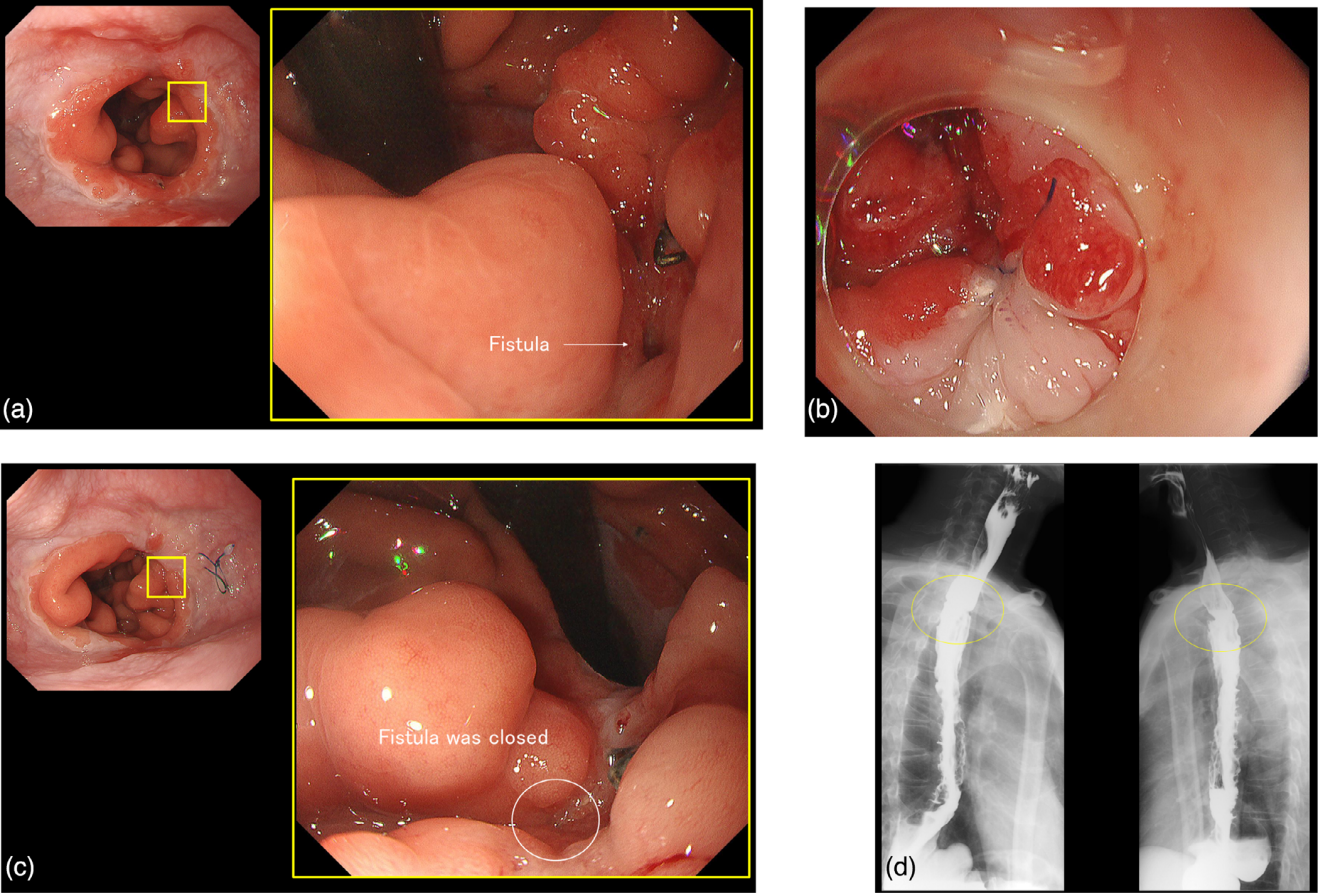
Images from Case 2 in which closure of the fistula was obtained with the endoscopic ligation technique (ELT). (a) A 2‐mm fistula located on the gastric tube side rather than at the anastomosis. The fistula could not be seen from the frontal view, so the endoscope was inverted. A stapler had been used during previous surgery in the vicinity of the fistula. (b) The fistula is sutured closed with one stitch by the ELT. (c) Two months later, observed under the same conditions as Figure 4a, the fistula near the stapled region has shrunk. (d) Postoperative esophagogastroduodenography performed 2 months after fistula closure with the ELT shows no leakage of contrast medium outside the lumen.

The fistula was located on the gastric tube side rather than at the anastomosis site (Figure 4a). Due to the presence of regenerative nodules caused by clips placed before the ELT was attempted, the fistula opening could not be directly visualized. Therefore, the endoscope was inverted to enable clear observation of the fistula. Markings were placed on the anal and oral sides of the fistula opening. A Z‐stitch was then placed to span the fistula opening in the anterior view, and the fistula was ligated with the ELT (Figure 4b).

Currently, 6 months have passed since the fistula closure via the ELT. The cough has completely resolved and the patient is eating without issues. Esophagography showed no contrast medium leakage from the fistula, and the fistula closure was observed endoscopically (Figure 4c,d).

## DISCUSSION

Gastrotracheal fistula occurs in 0.3% of cases after surgery for esophageal cancer.^4^ If there is a significant decline in respiratory function due to aspiration, surgery is indicated. Even in cases not requiring surgery, gastrotracheal fistula can lead to long‐term fasting requirements and chronic cough, which significantly reduce the quality of life.

The main treatment for gastrotracheal fistula is surgical. Common closure methods involve the placement of tissues, such as muscular flaps. However, surgery is invasive and causes cosmetic issues, while non‐surgical conservative treatments lead to closure in rare cases^5^, ^6^; thus, a conservative approach using endoscopy may be an option.^7^, ^8^, ^9^

We used the ELT to close refractory gastrotracheal fistulae. However, complete closure was only achieved in one of two cases. The difficulty in achieving closure of tracheoesophageal fistulas may be attributed to local factors such as 1) strong pressure exerted toward the fistula opening due to coughing, 2) poor blood flow in the fistula area, and 3) foreign bodies or infections in the fistula area.

While conventional clip‐based closure creates a suture‐like effect by bringing the mucosa together, the ELT allows for suturing through multiple layers (including submucosa and muscular layers) or full layers, depending on the needle type and technique. Even though over‐the‐scope clips have a strong grasping power, they do not provide complete full‐layer suturing.

Thus, the ELT may offer the potential for stronger and more sustainable closure than clip‐based methods. Therefore, if the only reason for fistula non‐closure is pressure from the trachea, robust closure using the ELT might be effective. However, if the reasons for non‐closure are poor blood flow, infection, or foreign bodies, surgical intervention might be necessary.

The ELT achieved complete closure in one case and temporary closure in the other. The fistula reopening in Case 1 could not be closed even through direct closure under visualization, which might be due to insufficient blood supply to the fistula area. The ELT may be useful in closing gastrointestinal tracheobronchial fistulas in select patients. However, further investigation is necessary.

The ELT also has potential for applications beyond fistula closure, such as suturing defects after endoscopic treatment, ligature for gastrointestinal perforations or bleeding, and even intracavity suturing. The limitations of clip‐based closure are associated with suture strength and sustainability; additionally, metallic foreign bodies remain, making clip‐based closure suitable for gastrointestinal procedures but less optimal for intracavity procedures. In contrast, the ELT offers a relatively simple alternative with potential for application within cavities due to the use of a suture that does not leave a metallic foreign body. Furthermore, in clip‐based closure, the clip remaining after the suture is placed may interfere with the next clip. If the ELT can be used to place some sutures, additional suturing with clips may be performed from that point on. Furthermore, if the thread is left uncut, it can be used as a traction thread to aid in the placement of the next suture.

Another endoscopic suturing method other than the ELT is the verbed suture, which is a continuous suture that requires changing the needle in the lumen to perform the next suture. However, changing the needle using an endoscope is difficult and time‐consuming. In the ELT, if the distance to the suture site is short enough, the needle can be changed manually by bringing the needle outside the body after one suture. Another advantage of the ELT is that if the thread breaks, it will not affect the entire sutured area, unlike continuous suturing.

The limitation of the ELT is that the distance to deliver the thread increases in tandem with the distance to the outside of the body. Therefore, the ligation area should ideally be located close to the outside of the body.

In conclusion, we developed a novel ELT with extracorporeal ligation using a flexible endoscope. This technique is useful as an aid in advanced flexible endoscopic surgery.

## CONFLICT OF INTEREST STATEMENT

There is no conflict of interest.

## Supporting information

A video showing the novel endoscopic ligation technique described in this report.
